# Supplementary material for: Long-Term Human Immune Reconstitution, T-Cell Development, and Immune Reactivity in Mice Lacking the Murine Major Histocompatibility Complex: Validation with Cellular and Gene Expression Profiles
Source: Cells. 2024 Oct 12;13(20):1686. doi: 10.3390/cells13201686 (PMC11506606; doi:10.3390/cells13201686)
Supplement: Supplementary file 1 [file cells-13-01686-s001.zip › cells-3225013-supplementary.pdf]

Supplementary Material

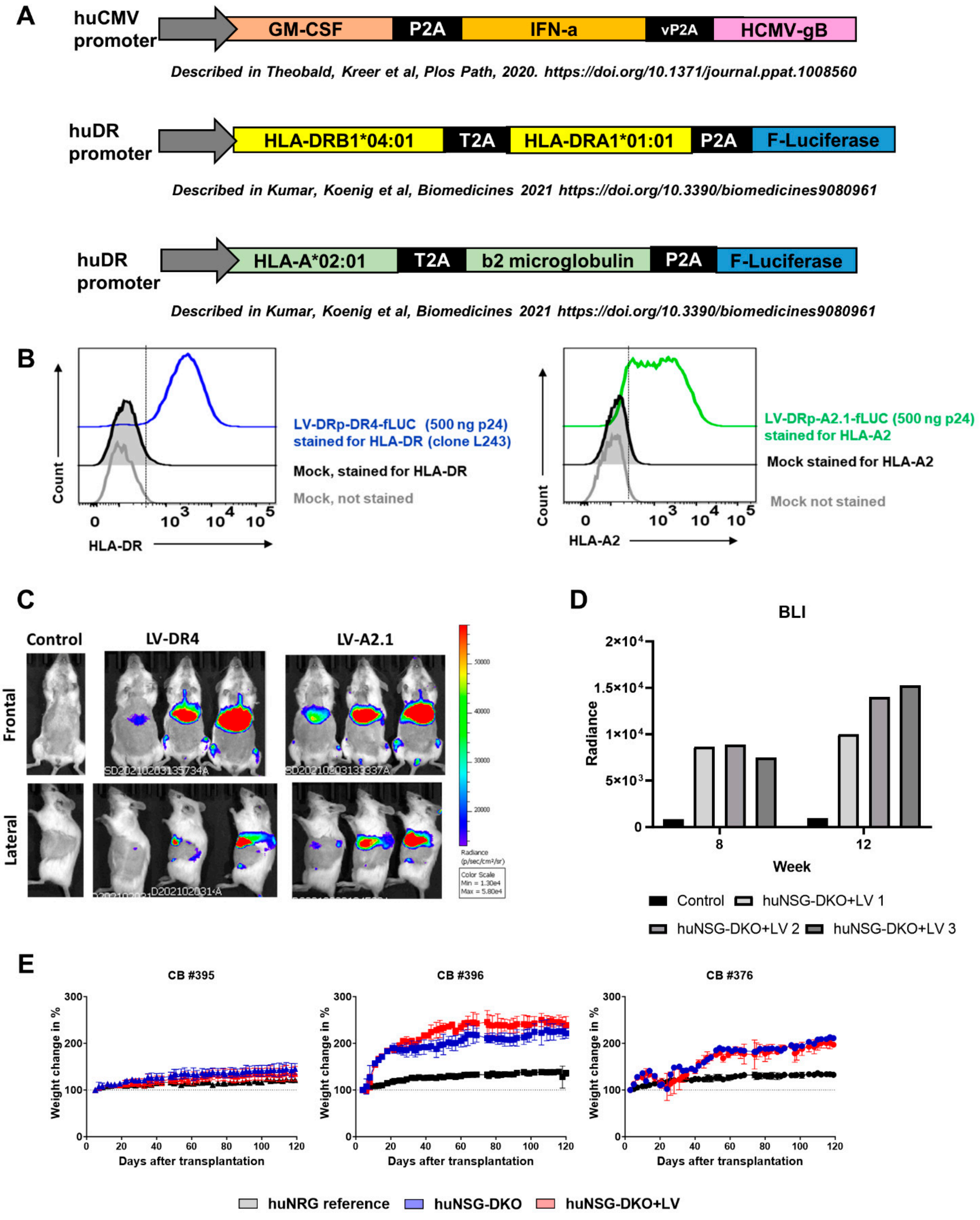

Figure S1. (A) Schematic representation of the multicistronic lentiviral vectors used in the study. (B) Detection of HLA-DR4 (upper panel) and HLA-A2.1 (lower panel) on 3T3 mouse fibroblasts transduced with lentiviral vectors. (C) Photographs corresponding to the detection of bioluminescent signals in non-humanized NSG-DKO mice injected with LV-DR4/fLuc and LV-A2.1/fLuc. The control mouse was injected with PBS. BLI measurements were performed at 8 weeks post-LV or PBS injection. (D) Quantification of BLI signal of humanized mice injected with LV-DR4/fLuc and LV-A2.1/fLuc. The control mouse (black bar) was injected with PBS. BLI measurements were performed at 8 and 12 weeks post-HCT. Quantification shows the results for three different mice injected with LV (gray bars). (E) Relative weight of huNRG (black), huNSG-DKO (blue), or NSG-DKO+LV (red) after reconstitution with CD34+ isolated cells from three different CB donors. HSCT was performed on day 0, and mice were sacrificed on day 120. The weight measured on day 0 was considered the 100% reference for each mouse. For the NSG-DKO+LV cohort (red), LVs were administered on week 1 (day 7) and week 8 (day 56) after HSCT.

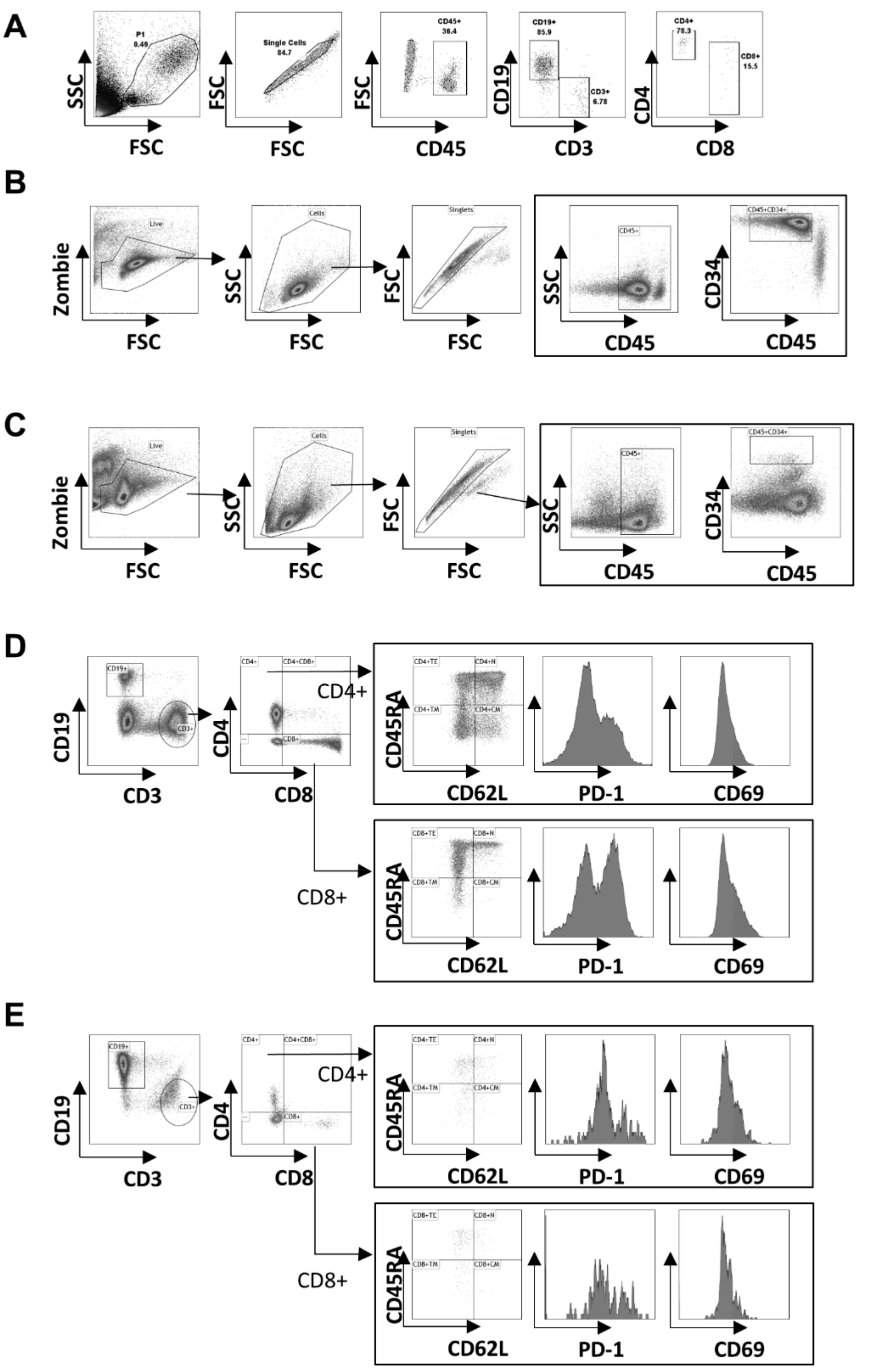

Figure S2. (A) Gating strategy for flow-cytometry analyses of human leukocytes in blood samples. Example showing blood 20 weeks post-HCT of a huNRG mouse as a reference. (B) Gating strategy for flow-cytometry analyses $CD45^+$ and $CD34^+$ cells. Example showing $CD34^+$ human cord blood. (C) Gating strategy for flow-cytometry analyses $CD45^+$ and $CD34^+$ cells. Example showing spleen of huNRG mouse as reference. (D) Gating strategy for flow-cytometry analyses and detection of PD-1 and CD69 activation markers. Example showing human PBMCs. (E) Gating strategy for flow-cytometry analyses and detection of PD-1 and CD69 activation markers. Example showing spleen of a huNRG mouse as reference.

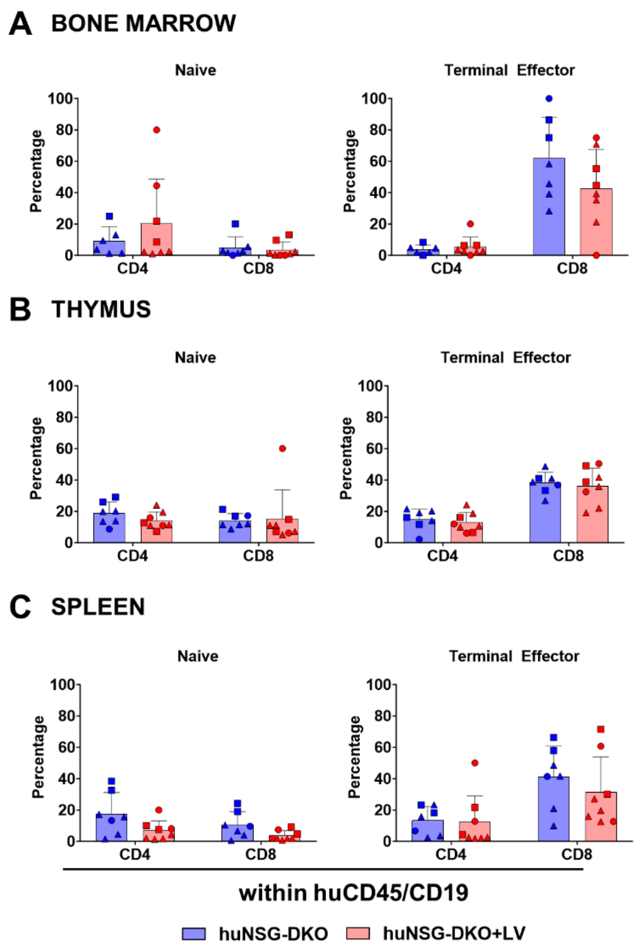

Figure S3. (A) Analysis of naive and terminal effector T-cell subtypes within huCD4+ and huCD8+ T-cells in bone marrow (in percentages). (B) analysis of naive and terminal effector T-cell subtypes within huCD4+ and huCD8+ T-cells in thymus (in percentages). (C) Analysis of naive and terminal effector T-cell subtypes within huCD4+ and huCD8+ T-cells in spleen (in percentages).

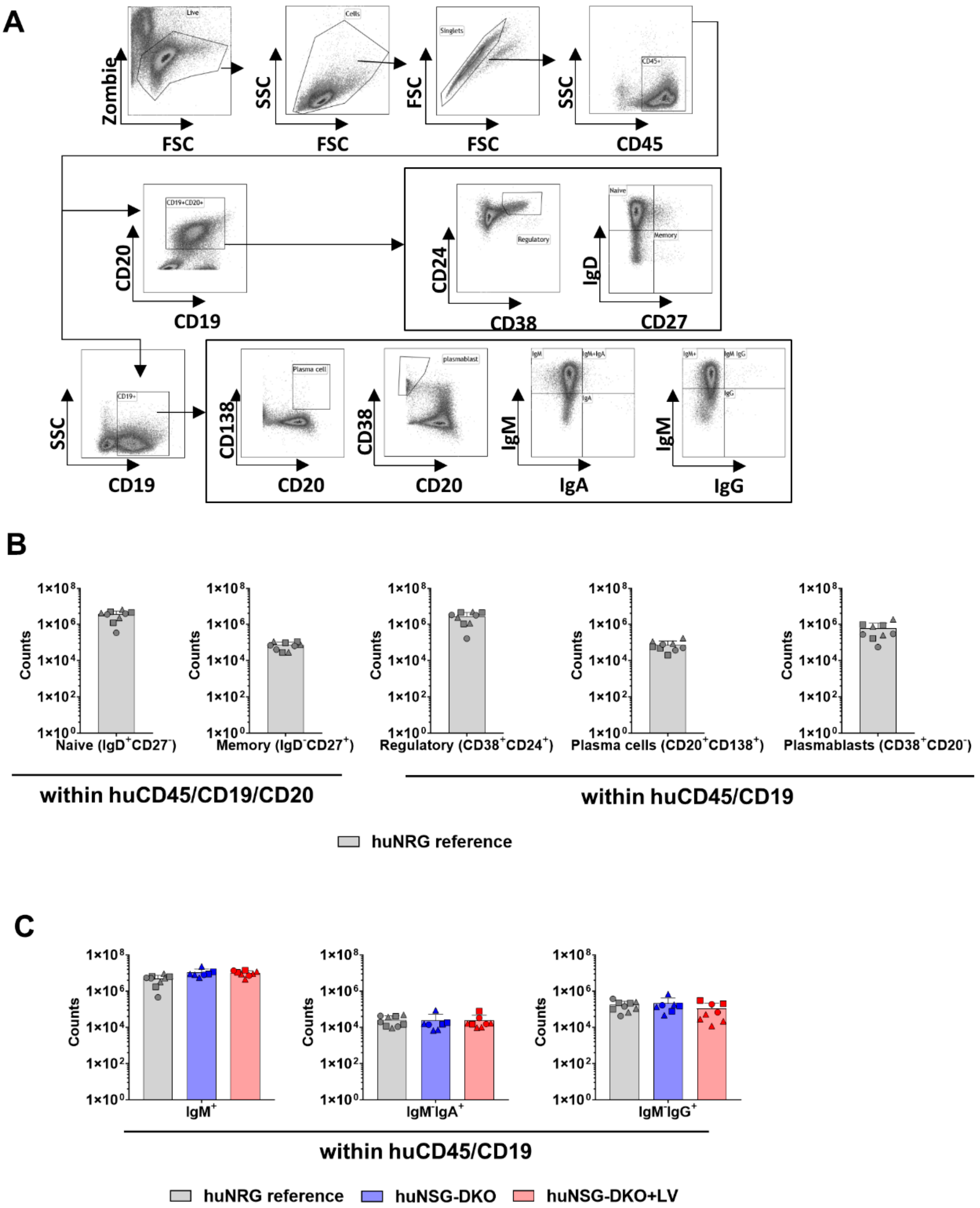

Figure S4. (A) Gating strategy for flow-cytometry analyses of B-cell subtypes. Example showing cells recovered from the spleen of a huNSG-DKO+LV mouse. (B) Analysis of B-cell subtypes in the spleens of huNRG reference mice. B-cell subtypes: naïve, memory, regulatory, plasma cells, and plasmablasts (in absolute cell counts, log scale). (C) Enumeration of IgM^{+}, IgM^{-}IgA^{+}, and IgM^{-}IgG^{+} B-cells in the spleen (in absolute cell counts, log scale).

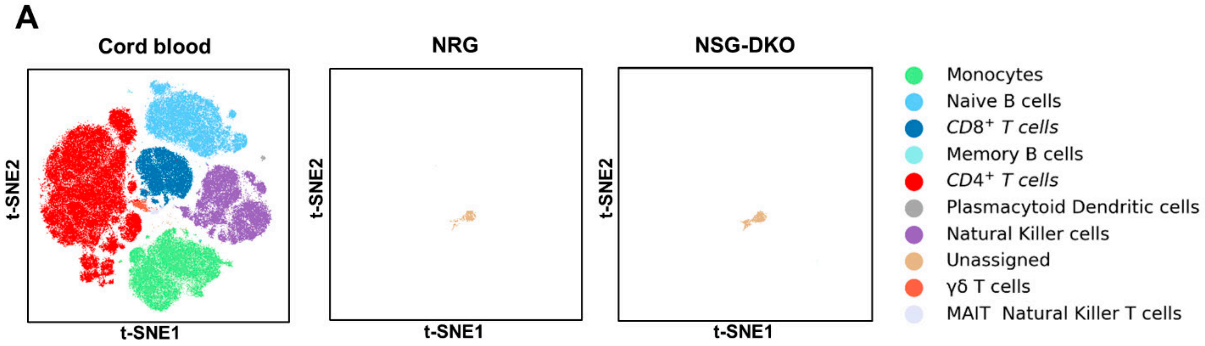

Figure S5. (A) CyTOF analyses to validate the methodology with reference samples: human cord blood (left panel), bone marrow of non-humanized NRG mouse (middle panel), and bone marrow of non-humanized NSG-DKO mouse (right panel).

Tables

Table S1. Information about mouse cohorts.

Donor	ID	HLA.A2.1	HLA.DRB1.04.01	Group	Male	Female	Depiction in Figures
1	CB #396	Positive	Negative	huNRG		3	Gray triangle
				huNSG-DKO	2	2	Blue triangle
				huNSG-DKO+LV	3	1	Red triangle
2	CB #395	Positive	Negative	huNRG	2	2	Gray square
				huNSG-DKO		2	Blue square
				huNSG-DKO+LV		2	Red square
3	CB #375	Positive	Negative	huNRG	2	2	Gray circle
				huNSG-DKO		1	Blue circle
				huNSG-DKO+LV		2	Red circle

Table S2. Antibodies used for FACS staining.

Antigen	Dye	Dilution	Clone	Cat. number	Source
CD3	BV510	1:100	UGHT1	#300448	Biolegend
CD45	Pacific blue	1:800	HI30	#304022	Biolegend
CD19	AF700	1:800	HIB19	#30225	Biolegend
CD62L	FITC	1:50	DREG-56	#304804	Biolegend
CD3	PerCp Cy5.5	1:100	SK7	#344808	Biolegend
PD-1	Pe-Dazzle	1:75	EH12.1	#565024	BD
CD45RA	PE-Cy7	1:75	HI100	#304126	Biolegend
CD34	APC	1:150	561	#343617	Biolegend
CD8	AF700	1:50	SK1	#344724	Biolegend
CD4	APC-Fire 750	1:75	SK3	#344638	Biolegend
CD69	BV510	1:75	FN50	#310936	Biolegend
CD45	BV785	1:150	HI30	# 304048	Biolegend
CD19	BUV737	1:50	SJ25C1	#564303	BD
CD20	FITC	1:75	2H7	#302304	Biolegend
CD38	PerCp Cy5.5	1:75	HIT2	#303522	Biolegend

IgA	PE	1:50	IS11-8E10	#130-113-476	Miltenyi
IgD	PE-Dazzle	1:75	IA6-2	#348240	Biolegend
CD27	PE-Cy7	1:75	O323	#302838	Biolegend
CD138	AF700	1:50	MI15	#356512	Biolegend
IgG	APC-Fire 750	1:50	M1310G05	#410724	Biolegend
IgM	BV421	1:100	MHM-88	#314514	Biolegend
CD24	BV605	1:75	ML5	#311124	Biolegend
CD45	BV785	1:150	HI30	#304048	Biolegend
CD19	BUV737	1:50	SJ25C1	#564303	BD
HLA-DR	APC	1:20	L243	#307609	Biolegend
HLA-A2	FITC	1:100	BB7.2	#343304	Biolegend

Table S3. Antibodies used for CyTOF staining.

Antigen	Metal	Dilution	Clone	Cat. number	Source
CD47	209Bi	1:50	CC2C6	#3209004B	Standard BioTools
CD5	194Pt	1:25	REA782	#130-124-324	Miltenyi
CD69	159Tb	1:100	FN50	#310939	BioLegend
Maxpar Direct Immune Profiling assay kit				#201334	Standard BioTools

Table S4. Mean, standard deviation, and group size of blood FACS data.

Figure	Tissue	Mouse age	Marker	Type	Group	Mean	Standard deviation	n
1 B	Blood	8	CD45	Percentage	huNRG	14.0	10.5	11
1 B	Blood	12	CD45	Percentage	huNRG	18.2	10.1	11
1 B	Blood	20	CD45	Percentage	huNRG	14.9	15.3	11
1 B	Blood	8	CD19	Percentage	huNRG	88.3	5.8	11
1 B	Blood	12	CD19	Percentage	huNRG	83.9	7.8	11
1 B	Blood	20	CD19	Percentage	huNRG	72.4	13.8	11
1 B	Blood	8	CD3	Percentage	huNRG	0.9	1.3	11
1 B	Blood	12	CD3	Percentage	huNRG	4.7	4.4	11
1 B	Blood	20	CD3	Percentage	huNRG	5.4	2.9	11
1 B	Blood	8	CD4	Percentage	huNRG	52.5	32.8	11
1 B	Blood	12	CD4	Percentage	huNRG	68.9	24.2	11
1 B	Blood	20	CD4	Percentage	huNRG	40.4	20.9	11
1 B	Blood	8	CD8	Percentage	huNRG	27.6	26.3	11
1 B	Blood	12	CD8	Percentage	huNRG	27.2	22.5	11
1 B	Blood	20	CD8	Percentage	huNRG	28.7	13.3	11
2 C	Blood	8	CD45	Percentage	huNSG-DKO	50.6	32.9	7
2 C	Blood	8	CD45	Percentage	huNSG-DKO+LV	53.3	32.2	8
2 C	Blood	12	CD45	Percentage	huNSG-DKO	58.1	8.7	7
2 C	Blood	12	CD45	Percentage	huNSG-DKO+LV	56.8	9.4	8
2 C	Blood	20	CD45	Percentage	huNSG-DKO	57.9	18.1	7
2 C	Blood	20	CD45	Percentage	huNSG-DKO+LV	59.4	19.4	8

2 C	Blood	8	CD19	Percentage	huNSG-DKO	92.6	4.8	7
2 C	Blood	8	CD19	Percentage	huNSG-DKO+LV	90.8	3.1	8
2 C	Blood	12	CD19	Percentage	huNSG-DKO	89.8	3.2	7
2 C	Blood	12	CD19	Percentage	huNSG-DKO+LV	88.9	2.9	8
2 C	Blood	20	CD19	Percentage	huNSG-DKO	82.6	10.0	7
2 C	Blood	20	CD19	Percentage	huNSG-DKO+LV	74.2	16.6	8
2 C	Blood	8	CD3	Percentage	huNSG-DKO	0.2	0.2	7
2 C	Blood	8	CD3	Percentage	huNSG-DKO+LV	0.3	0.1	8
2 C	Blood	12	CD3	Percentage	huNSG-DKO	1.6	1.0	7
2 C	Blood	12	CD3	Percentage	huNSG-DKO+LV	1.7	1.2	8
2 C	Blood	20	CD3	Percentage	huNSG-DKO	7.5	10.7	7
2 C	Blood	20	CD3	Percentage	huNSG-DKO+LV	14.2	18.4	8
2 C	Blood	8	CD4	Percentage	huNSG-DKO	42.8	38.6	7
2 C	Blood	8	CD4	Percentage	huNSG-DKO+LV	46.7	28.9	8
2 C	Blood	12	CD4	Percentage	huNSG-DKO	42.4	7.8	7
2 C	Blood	12	CD4	Percentage	huNSG-DKO+LV	44.7	16.7	8
2 C	Blood	20	CD4	Percentage	huNSG-DKO	49.2	13.6	7
2 C	Blood	20	CD4	Percentage	huNSG-DKO+LV	45.9	24.1	8
2 C	Blood	8	CD8	Percentage	huNSG-DKO	11.1	14.3	7
2 C	Blood	8	CD8	Percentage	huNSG-DKO+LV	29.1	27.3	8
2 C	Blood	12	CD8	Percentage	huNSG-DKO	41.8	6.6	7
2 C	Blood	12	CD8	Percentage	huNSG-DKO+LV	39	14	8
2 C	Blood	20	CD8	Percentage	huNSG-DKO	28.3	12.5	7
2 C	Blood	20	CD8	Percentage	huNSG-DKO+LV	27.3	9.7	8

Table S5. Statistical analysis comparing FACS data of blood between huNSG-DKO and huNSG-DKO+LV.

Figure	Tissue	Mouse age	Marker	Type	p value
2 C	Blood	8	CD45	Percentage	0.88
2 C	Blood	12	CD45	Percentage	0.79
2 C	Blood	20	CD45	Percentage	0.88
2 C	Blood	8	CD19	Percentage	0.43
2 C	Blood	12	CD19	Percentage	0.58
2 C	Blood	20	CD19	Percentage	0.25
2 C	Blood	8	CD3	Percentage	0.28
2 C	Blood	12	CD3	Percentage	0.98
2 C	Blood	20	CD3	Percentage	0.40
2 C	Blood	8	CD4	Percentage	0.83
2 C	Blood	12	CD4	Percentage	0.74
2 C	Blood	20	CD4	Percentage	0.75
2 C	Blood	8	CD8	Percentage	0.13
2 C	Blood	12	CD8	Percentage	0.62

2 C	Blood	20	CD8	Percentage	

Table S6. Mean, standard deviation, and group size of tissue FACS data.

Figure	Tissue	Marker	Type	Group	Mean	Standard deviation	n
1 C	Bone marrow	CD45	Counts	huNRG	21722866	10072002	11
1 C	Bone marrow	CD34	Counts	huNRG	1036343	921003	11
1 C	Bone marrow	CD3	Counts	huNRG	518574	799596	11
1 C	Bone marrow	CD4	Counts	huNRG	272189	540539	11
1 C	Bone marrow	CD8	Counts	huNRG	126650	237705	11
1 C	Bone marrow	DP	Counts	huNRG	54487	86049	11
1 D	Thymus	CD45	Counts	huNRG	5625366	7199294	11
1 D	Thymus	CD34	Counts	huNRG	8508	11667	11
1 D	Thymus	CD3	Counts	huNRG	2281408	3945192	11
1 D	Thymus	CD4	Counts	huNRG	714080	1379226	11
1 D	Thymus	CD8	Counts	huNRG	1058000	1553740	11
1 D	Thymus	DP	Counts	huNRG	230326	567196	11
1 E	Spleen	CD45	Counts	huNRG	8107724	4041508	9
1 E	Spleen	CD34	Counts	huNRG	97419	101295	9
1 E	Spleen	CD3	Counts	huNRG	857846	378271	9
1 E	Spleen	CD4	Counts	huNRG	180058	204384	9
1 E	Spleen	CD8	Counts	huNRG	137590	91127	9
1 E	Spleen	DP	Counts	huNRG	46395	97459	9
2 D	Bone marrow	CD45	Counts	huNSG-DKO	23198987	10251075	7
2 D	Bone marrow	CD45	Counts	huNSG-DKO+LV	28251568	7453254	8
2 D	Bone marrow	CD34	Counts	huNSG-DKO	1046011	817036	7
2 D	Bone marrow	CD34	Counts	huNSG-DKO+LV	839373	773635	8
2 D	Bone marrow	CD3	Counts	huNSG-DKO	203394	431284	7
2 D	Bone marrow	CD3	Counts	huNSG-DKO+LV	752338	1061005	8
2 D	Bone marrow	CD4	Counts	huNSG-DKO	134420	316673	7
2 D	Bone marrow	CD4	Counts	huNSG-DKO+LV	391486	539548	8
2 D	Bone marrow	CD8	Counts	huNSG-DKO	38447	59878	7

2 D	Bone marrow	CD8	Counts	huNSG-DKO+LV	216604	403398	8
2 D	Bone marrow	DP	Counts	huNSG-DKO	12313	27463	7
2 D	Bone marrow	DP	Counts	huNSG-DKO+LV	61190	101785	8
2 E	Thymus	CD45	Counts	huNSG-DKO	10101991	10983519	7
2 E	Thymus	CD45	Counts	huNSG-DKO+LV	8462185	7151289	8
2 E	Thymus	CD34	Counts	huNSG-DKO	13404	14933	7
2 E	Thymus	CD34	Counts	huNSG-DKO+LV	8376	8084	8
2 E	Thymus	CD3	Counts	huNSG-DKO	4919346	5311069	7
2 E	Thymus	CD3	Counts	huNSG-DKO+LV	3812112	4180081	8
2 E	Thymus	CD4	Counts	huNSG-DKO	206029	219464	7
2 E	Thymus	CD4	Counts	huNSG-DKO+LV	526215	587569	8
2 E	Thymus	CD8	Counts	huNSG-DKO	2420489	2565549	7
2 E	Thymus	CD8	Counts	huNSG-DKO+LV	1774081	1858031	8
2 E	Thymus	DP	Counts	huNSG-DKO	946089	1162670	7
2 E	Thymus	DP	Counts	huNSG-DKO+LV	682349	886331	8
2 F	Spleen	CD45	Counts	huNSG-DKO	16160786	8977472	7
2 F	Spleen	CD45	Counts	huNSG-DKO+LV	15771427	6761737	8
2 F	Spleen	CD34	Counts	huNSG-DKO	223570	214696	7
2 F	Spleen	CD34	Counts	huNSG-DKO+LV	101874	161542	8
2 F	Spleen	CD3	Counts	huNSG-DKO	667749	438389	7
2 F	Spleen	CD3	Counts	huNSG-DKO+LV	1069744	729408	8
2 F	Spleen	CD4	Counts	huNSG-DKO	206029	219464	7
2 F	Spleen	CD4	Counts	huNSG-DKO+LV	526215	587569	8
2 F	Spleen	CD8	Counts	huNSG-DKO	148287	88492	7
2 F	Spleen	CD8	Counts	huNSG-DKO+LV	239907	181219	8
2 F	Spleen	DP	Counts	huNSG-DKO	17356	12933	7
2 F	Spleen	DP	Counts	huNSG-DKO+LV	59977	69083	8
3 A	Bone marrow	CD4 CM	Percentage	huNSG-DKO	16.2	5.2	6
3 A	Bone marrow	CD4 CM	Percentage	huNSG-DKO+LV	18.1	16.4	8
3 A	Bone marrow	CD4 EM	Percentage	huNSG-DKO	71.1	15.7	6
3 A	Bone marrow	CD4 EM	Percentage	huNSG-DKO+LV	56.2	36.2	8
3 A	Bone marrow	CD8 CM	Percentage	huNSG-DKO	1.8	1.9	7
3 A	Bone marrow	CD8 CM	Percentage	huNSG-DKO+LV	3.5	5.0	8
3 A	Bone marrow	CD8 EM	Percentage	huNSG-DKO	31.4	27.1	7
3 A	Bone marrow	CD8 EM	Percentage	huNSG-DKO+LV	43.9	17.7	8

3 B	Thymus	CD4 CM	Percentage	huNSG-DKO	15.7	5.2	7
3 B	Thymus	CD4 CM	Percentage	huNSG-DKO+LV	17.2	4.9	8
3 B	Thymus	CD4 EM	Percentage	huNSG-DKO	50.6	7.9	7
3 B	Thymus	CD4 EM	Percentage	huNSG-DKO+LV	55.6	6.9	8
3 B	Thymus	CD8 CM	Percentage	huNSG-DKO	2.8	1.5	7
3 B	Thymus	CD8 CM	Percentage	huNSG-DKO+LV	2.9	1.9	8
3 B	Thymus	CD8 EM	Percentage	huNSG-DKO	44.9	7.8	7
3 B	Thymus	CD8 EM	Percentage	huNSG-DKO+LV	45.6	20.7	8
3 C	Spleen	CD4 CM	Percentage	huNSG-DKO	20.9	9.8	7
3 C	Spleen	CD4 CM	Percentage	huNSG-DKO+LV	28.7	11.3	8
3 C	Spleen	CD4 EM	Percentage	huNSG-DKO	48.2	18.5	7
3 C	Spleen	CD4 EM	Percentage	huNSG-DKO+LV	52.2	17.3	8
3 C	Spleen	CD8 CM	Percentage	huNSG-DKO	7.9	5.4	7
3 C	Spleen	CD8 CM	Percentage	huNSG-DKO+LV	8.1	7.1	8
3 C	Spleen	CD8 EM	Percentage	huNSG-DKO	40.5	24.0	7
3 C	Spleen	CD8 EM	Percentage	huNSG-DKO+LV	56.9	21.6	8
3 D	Bone marrow	PD-1 on CD4	MFI	huNSG-DKO	174410	107859	6
3 D	Bone marrow	PD-1 on CD4	MFI	huNSG-DKO+LV	147673	111433	8
3 D	Bone marrow	PD-1 on CD8	MFI	huNSG-DKO	85517	87129	7
3 D	Bone marrow	PD-1 on CD8	MFI	huNSG-DKO+LV	103579	79255	8
3 D	Bone marrow	CD69 on CD4	MFI	huNSG-DKO	12055	4031	6
3 D	Bone marrow	CD69 on CD4	MFI	huNSG-DKO+LV	14995	4015	8
3 D	Bone marrow	CD69 on CD8	MFI	huNSG-DKO	12539	6653	7
3 D	Bone marrow	CD69 on CD8	MFI	huNSG-DKO+LV	9954	3097	8
3 E	Thymus	PD-1 on CD4	MFI	huNSG-DKO	31400	18770	7
3 E	Thymus	PD-1 on CD4	MFI	huNSG-DKO+LV	35727	24062	8
3 E	Thymus	PD-1 on CD8	MFI	huNSG-DKO	51405	25583	7
3 E	Thymus	PD-1 on CD8	MFI	huNSG-DKO+LV	73208	64109	8
3 E	Thymus	CD69 on CD4	MFI	huNSG-DKO	14060	3863	7
3 E	Thymus	CD69 on CD4	MFI	huNSG-DKO+LV	13336	6540	8
3 E	Thymus	CD69 on CD8	MFI	huNSG-DKO	16371	7092	7
3 E	Thymus	CD69 on CD8	MFI	huNSG-DKO+LV	17010	8596	8
3 F	Spleen	PD-1 on CD4	MFI	huNSG-DKO	46387	45243	7
3 F	Spleen	PD-1 on CD4	MFI	huNSG-DKO+LV	208139	254346	8
3 F	Spleen	PD-1 on CD8	MFI	huNSG-DKO	33598	30933	7
3 F	Spleen	PD-1 on CD8	MFI	huNSG-DKO+LV	75615	56216	8
3 F	Spleen	CD69 on CD4	MFI	huNSG-DKO	7451	1579	7

3 F	Spleen	CD69 on CD4	MFI	huNSG-DKO+LV	8440	2026	8
3 F	Spleen	CD69 on CD8	MFI	huNSG-DKO	4823	538	7
3 F	Spleen	CD69 on CD8	MFI	huNSG-DKO+LV	5063	1818	8
3 G	Spleen	Naive B-cells	Counts	huNSG-DKO	8024367	4878424	7
3 G	Spleen	Naive B-cells	Counts	huNSG-DKO+LV	7944084	2533333	8
3 G	Spleen	Memory B-cells	Counts	huNSG-DKO	116292	63031	7
3 G	Spleen	Memory B-cells	Counts	huNSG-DKO+LV	75674	41744	8
3 G	Spleen	Regulatory B-cells	Counts	huNSG-DKO	4492862	2369896	7
3 G	Spleen	Regulatory B-cells	Counts	huNSG-DKO+LV	3799313	2868174	8
3 G	Spleen	Plasma cells	Counts	huNSG-DKO	105376	102043	7
3 G	Spleen	Plasma cells	Counts	huNSG-DKO+LV	46564	24347	8
3 G	Spleen	Plasmablasts	Counts	huNSG-DKO	1675190	1046565	7
3 G	Spleen	Plasmablasts	Counts	huNSG-DKO+LV	886585	418832	8
S3 A	Bone marrow	CD4 N	Percentage	huNSG-DKO	9.0	9.1	6
S3 A	Bone marrow	CD4 N	Percentage	huNSG-DKO+LV	20.3	28.4	8
S3 A	Bone marrow	CD4 TE	Percentage	huNSG-DKO	3.7	3.0	6
S3 A	Bone marrow	CD4 TE	Percentage	huNSG-DKO+LV	5.5	6.2	8
S3 A	Bone marrow	CD8 N	Percentage	huNSG-DKO	5.0	6.9	7
S3 A	Bone marrow	CD8 N	Percentage	huNSG-DKO+LV	3.5	5.0	8
S3 A	Bone marrow	CD8 TE	Percentage	huNSG-DKO	61.8	26.3	7
S3 A	Bone marrow	CD8 TE	Percentage	huNSG-DKO+LV	42.7	24.9	8
S3 B	Thymus	CD4 N	Percentage	huNSG-DKO	18.8	7.3	7
S3 B	Thymus	CD4 N	Percentage	huNSG-DKO+LV	14.1	5.4	8
S3 B	Thymus	CD4 TE	Percentage	huNSG-DKO	15.0	6.6	7
S3 B	Thymus	CD4 TE	Percentage	huNSG-DKO+LV	13.0	6.3	8
S3 B	Thymus	CD8 N	Percentage	huNSG-DKO	14.2	4.4	7
S3 B	Thymus	CD8 N	Percentage	huNSG-DKO+LV	15.3	18.4	8
S3 B	Thymus	CD8 TE	Percentage	huNSG-DKO	38.1	6.8	7
S3 B	Thymus	CD8 TE	Percentage	huNSG-DKO+LV	36.2	11.4	8
S3 C	Spleen	CD4 N	Percentage	huNSG-DKO	17.6	13.6	7
S3 C	Spleen	CD4 N	Percentage	huNSG-DKO+LV	6.9	6.2	8
S3 C	Spleen	CD4 TE	Percentage	huNSG-DKO	13.3	9.0	7
S3 C	Spleen	CD4 TE	Percentage	huNSG-DKO+LV	12.3	16.8	8
S3 C	Spleen	CD8 N	Percentage	huNSG-DKO	10.6	8.3	7
S3 C	Spleen	CD8 N	Percentage	huNSG-DKO+LV	3.7	3.1	8

S3 C	Spleen	CD8 TE	Percentage	huNSG-DKO	41.0	19.8	7
S3 C	Spleen	CD8 TE	Percentage	huNSG-DKO+LV	31.3	22.6	8
S4 B	Spleen	Naïve B-cells	Counts	huNRG	3443476	1891552	9
S4 B	Spleen	Memory B-cells	Counts	huNRG	69341	32531	9
S4 B	Spleen	Regulatory B-cells	Counts	huNRG	2742742	1673443	9
S4 B	Spleen	Plasma cells	Counts	huNRG	73016	46023	9
S4 B	Spleen	Plasmablasts	Counts	huNRG	602746	567353	9
S4 C	Spleen	IgM	Counts	huNRG	4808170	2637894	9
S4 C	Spleen	IgM	Counts	huNSG-DKO	10601939	5870349	7
S4 C	Spleen	IgM	Counts	huNSG-DKO+LV	10005255	3312436	8
S4 C	Spleen	IgA	Counts	huNRG	26131	15944	9
S4 C	Spleen	IgA	Counts	huNSG-DKO	23522	28684	7
S4 C	Spleen	IgA	Counts	huNSG-DKO+LV	24211	22648	8
S4 C	Spleen	IgG	Counts	huNRG	169995	105892	9
S4 C	Spleen	IgG	Counts	huNSG-DKO	204569	216784	7
S4 C	Spleen	IgG	Counts	huNSG-DKO+LV	108820	106782	8

Table S7. Statistical analysis comparing FACS data of tissues between huNSG-DKO and huNSG-DKO+LV.

Figure	Tissue	Marker	Type	p value
2 D	Bone marrow	CD45	Counts	0.32
2 D	Bone marrow	CD34	Counts	0.77
2 D	Bone marrow	CD3	Counts	0.08
2 D	Bone marrow	CD4	Counts	0.22
2 D	Bone marrow	CD8	Counts	0.43
2 D	Bone marrow	DP	Counts	0.12
2 E	Thymus	CD45	Counts	0.97
2 E	Thymus	CD34	Counts	0.31
2 E	Thymus	CD3	Counts	0.74
2 E	Thymus	CD4	Counts	0.55
2 E	Thymus	CD8	Counts	0.80
2 E	Thymus	DP	Counts	0.74
2 F	Spleen	CD45	Counts	0.99
2 F	Spleen	CD34	Counts	0.16
2 F	Spleen	CD3	Counts	0.21
2 F	Spleen	CD4	Counts	0.43
2 F	Spleen	CD8	Counts	0.25
2 F	Spleen	DP	Counts	0.17
3 A	Bone marrow	CD4 CM	Percentage	0.77
3 A	Bone marrow	CD4 EM	Percentage	0.32
3 A	Bone marrow	CD8 CM	Percentage	0.35
3 A	Bone marrow	CD8 EM	Percentage	0.32

3 B	Thymus	CD4 CM	Percentage	0.55
3 B	Thymus	CD4 EM	Percentage	0.22
3 B	Thymus	CD8 CM	Percentage	0.92
3 B	Thymus	CD8 EM	Percentage	0.94
3 C	Spleen	CD4 CM	Percentage	0.50
3 C	Spleen	CD4 EM	Percentage	0.40
3 C	Spleen	CD8 CM	Percentage	0.09
3 C	Spleen	CD8 EM	Percentage	0.10
3 D	Bone marrow	PD-1 on CD4	MFI	0.66
3 D	Bone marrow	PD-1 on CD8	MFI	0.68
3 D	Bone marrow	CD69 on CD4	MFI	0.20
3 D	Bone marrow	CD69 on CD8	MFI	0.37
3 E	Thymus	PD-1 on CD4	MFI	0.70
3 E	Thymus	PD-1 on CD8	MFI	0.40
3 E	Thymus	CD69 on CD4	MFI	0.80
3 E	Thymus	CD69 on CD8	MFI	0.88
3 F	Spleen	PD-1 on CD4	MFI	0.12
3 F	Spleen	PD-1 on CD8	MFI	0.10
3 F	Spleen	CD69 on CD4	MFI	0.31
3 F	Spleen	CD69 on CD8	MFI	0.73
3 G	Spleen	Naive B-cells	Counts	0.83
3 G	Spleen	Memory B-cells	Counts	0.20
3 G	Spleen	Regulatory B-cells	Counts	0.49
3 G	Spleen	Plasma cells	Counts	0.37
3 G	Spleen	Plasmablasts	Counts	0.13
S3 A	Bone marrow	CD4 N	Percentage	0.32
S3 A	Bone marrow	CD4 TE	Percentage	0.48
S3 A	Bone marrow	CD8 N	Percentage	0.65
S3 A	Bone marrow	CD8 TE	Percentage	0.17
S3 B	Thymus	CD4 N	Percentage	0.19
S3 B	Thymus	CD4 TE	Percentage	0.58
S3 B	Thymus	CD8 N	Percentage	0.86
S3 B	Thymus	CD8 TE	Percentage	0.70
S3 C	Spleen	CD4 N	Percentage	0.32
S3 C	Spleen	CD4 TE	Percentage	0.45
S3 C	Spleen	CD8 N	Percentage	0.06
S3 C	Spleen	CD8 TE	Percentage	0.07
S4 C	Spleen	IgM	Counts	0.94
S4 C	Spleen	IgA	Counts	0.68
S4 C	Spleen	IgG	Counts	0.14

Table S8. Mean, standard deviation, and group size of CyTOF data.

Figure	Tissue	Marker	Type	Group	Mean	Standard deviation	n
4 D	Bone marrow	Monocytes	Counts	huNRG	2758	715	3
4 D	Bone marrow	CD4 T-cells	Counts	huNRG	293	237	3
4 D	Bone marrow	CD8 T-cells	Counts	huNRG	82	48	3
4 D	Bone marrow	Plasmacytoid dendritic cells	Counts	huNRG	1353	173	3
4 D	Bone marrow	Myeloid dendritic cells	Counts	huNRG	496	172	3
4 D	Bone marrow	Natural killer cells	Counts	huNRG	168	103	3
4 D	Bone marrow	γδ T-cells	Counts	huNRG	20	13	3
5 C	Bone marrow	Monocytes	Counts	huNSG-DKO	3543	459	4
5 C	Bone marrow	Monocytes	Counts	huNSG-DKO+LV	4717	2369	4
5 C	Bone marrow	CD4 T-cells	Counts	huNSG-DKO	999	1625	4
5 C	Bone marrow	CD4 T-cells	Counts	huNSG-DKO+LV	3054	2708	4
5 C	Bone marrow	CD8 T-cells	Counts	huNSG-DKO	229	234	4
5 C	Bone marrow	CD8 T-cells	Counts	huNSG-DKO+LV	1391	1992	4
5 C	Bone marrow	Plasmacytoid dendritic cells	Counts	huNSG-DKO	1318	210	4
5 C	Bone marrow	Plasmacytoid dendritic cells	Counts	huNSG-DKO+LV	1465	228	4
5 C	Bone marrow	Myeloid dendritic cells	Counts	huNSG-DKO	518	110	4
5 C	Bone marrow	Myeloid dendritic cells	Counts	huNSG-DKO+LV	802	328	4
5 C	Bone marrow	Natural killer cells	Counts	huNSG-DKO	158	57	4
5 C	Bone marrow	Natural killer cells	Counts	huNSG-DKO+LV	346	320	4
5 C	Bone marrow	γδ T-cells	Counts	huNSG-DKO	72	77	4
5 C	Bone marrow	γδ T-cells	Counts	huNSG-DKO+LV	165	147	4
5 D	Bone marrow	HLA-DR on CD4 T-cells	MFI	huNSG-DKO	0.20	0.06	4
5 D	Bone marrow	HLA-DR on CD4 T-cells	MFI	huNSG-DKO+LV	0.22	0.04	4
5 D	Bone marrow	HLA-DR on CD8 T-cells	MFI	huNSG-DKO	0.18	0.08	4

5 D	Bone marrow	HLA-DR on CD8 T-cells	MFI	huNSG-DKO+LV	0.24	0.05	4
5 D	Bone marrow	HLA-DR on γδ T-cells	MFI	huNSG-DKO	0.16	0.08	4
5 D	Bone marrow	HLA-DR on γδ T-cells	MFI	huNSG-DKO+LV	0.16	0.02	4

Table S9. Statistical analysis comparing CyTOF data between huNSG-DKO and huNSG-DKO+LV.

Figure	Tissue	Marker	Type	p value
5 C	Bone marrow	Monocytes	Counts	0.77
5 C	Bone marrow	CD4 T-cells	Counts	0.17
5 C	Bone marrow	CD8 T-cells	Counts	0.11
5 C	Bone marrow	Plasmacytoid dendritic cells	Counts	0.39
5 C	Bone marrow	Myeloid dendritic cells	Counts	0.14
5 C	Bone marrow	Natural killer cells	Counts	0.40
5 C	Bone marrow	γδ T-cells	Counts	0.34
5 D	Bone marrow	HLA-DR on CD4 T-cells	MFI	0.74
5 D	Bone marrow	HLA-DR on CD8 T-cells	MFI	0.29
5 D	Bone marrow	HLA-DR on γδ T-cells	MFI	0.93
